# The Relationship Between Nutrition and Atherosclerosis

**DOI:** 10.3389/fbioe.2021.635504

**Published:** 2021-04-19

**Authors:** Taotao Wei, Junnan Liu, Demei Zhang, Xiaomei Wang, Guangling Li, Ruchao Ma, Gang Chen, Xin Lin, Xueya Guo

**Affiliations:** ^1^Department of Cardiology, Lanzhou University Second Hospital, Lanzhou University, Lanzhou, China; ^2^Lanzhou University Second Hospital, The Second Clinical Medical College of Lanzhou University, Lanzhou, China

**Keywords:** atherosclerosis, nutrients, antioxidants, vitamin, omega-3

## Abstract

Atherosclerosis is the basic pathological process of many diseases, such as coronary atherosclerosis and stroke. Nutrients can affect the occurrence and development of atherosclerosis. At present, in nutrition science, the research on atherosclerosis focuses on which nutrients play an important role in its prevention strategy, and what are the possible mechanisms of its action. In the current study, the process of atherosclerosis can be affected by adjusting the proportion of nutrients in the diet. In this review, we pay attention to the effects of phytosterols, omega-3-polyunsaturated fatty acids, polyphenol, vitamin, and other nutrients on atherosclerosis, pay attention to their current epidemiological status, current nutritional research results, and prevention or a possible mechanism to reduce the risk of development of atherosclerosis. So that more personalized nutritional approaches may be more effective in terms of nutritional intervention responses to atherosclerosis.

## Introduction

Due to population growth, population aging, and disease epidemiological changes, the number of deaths from cardiovascular diseases (CVDs) is increasing in worldwide. From 1990 to 2013, the number of deaths due to CVD increased by 41% in the worldwide. In 2016, CVD caused about 17.6 million deaths worldwide, an increase of 14.5% compared with 2006. CVD cause huge health and economic burdens in the United States and globally (Benjamin et al., 2019).

Atherosclerosis is defined as a chronic inflammatory disease. The infiltration and retention of lipoproteins in the arterial wall is a key initiation event that triggers an inflammatory response and promotes the development of atherosclerosis. Blood lipids are transported in the form of lipoproteins in the blood circulation. Studies have found that the increase in plasma low-density lipoprotein (LDL) levels and the decrease in high-density lipoprotein (HDL) levels are positively correlated with the incidence of atherosclerosis. After being oxidized and modified by arterial wall cells, LDL can promote the formation of atherosclerotic plaques. At present, oxidized LDL (ox-LDL) is considered to be an important atherosclerotic factor and a major factor that causes damage to endothelial cells and smooth muscle cells. Ox-LDL cannot be recognized by normal LDL receptors, but easily recognized by scavenger receptors of macrophages and quickly taken up, which promotes the formation of foam cells by macrophages. On the contrary, HDL remove cholesterol from the arterial wall through the cholesterol reverse transport mechanism, and prevent the occurrence of atherosclerosis. In addition, HDL also has antioxidant effects, can prevent the oxidation of LDL, and can competitively inhibit the receptors of LDL and endothelial cells.

The pathogenesis of atherosclerosis has not been elucidated. The injure of endothelial cells caused by various reasons leads to endothelial dysfunction, promotes the modification of lipoproteins and the infiltration of monocytes into inner subcutaneous space. The increased plasma lipoprotein is deposited on the arterial intima, causing connective tissue hyperplasia, thickening and hardening of the arterial wall, and then necrosis of the connective tissue to form atherosclerosis. Endothelial cells are damaged due to various reasons, so that plasma components include lipoprotein deposits on the inner membrane, causing platelets to adhere, aggregate, release various active substances [monocyte chemoattractant protein-1, interleukin (IL)-8, intercellular adhesion molecule-1 (ICAM-1), vascular adhesion molecule-1 (VCAM-1), E-selectin, and P-selectin], attract monocytes to aggregate, adhere to the endothelium, and migrate to the subendothelial tissue of the blood vessel, and combine with oxidized lipoproteins to form mononuclear cells. At the same time, the active substance activates the smooth muscle cells of the arterial media to migrate into the intima to form smooth muscle-derived foam cells. Finally, the proliferating smooth muscle cells synthesize extracellular matrix such as collagen and proteoglycan to thicken and harden the intima of the disease, promote plaque formation, and accelerate the development of atherosclerosis.

Studies have found that NO can prevent the expression of pro-inflammatory factors, such as nuclear factor NF-κB (NF-kB) and adhesion molecules (monocyte chemoattractant protein-1, ICAM-1, VCAM-1, E-selectin, and P-selectin) (Badimón et al., 2009; Zhu et al., 2018). There are also studies show a large amount of reactive oxygen species (ROS) can mediate vascular endothelial dysfunction by promoting the action of superoxide dismutase, weaken the antioxidant capacity in cells, cause lipid peroxidation and DNA damage, and cause atherosclerosis (Förstermann et al., 2017; Zhu et al., 2018).

**FIGURE 1:**
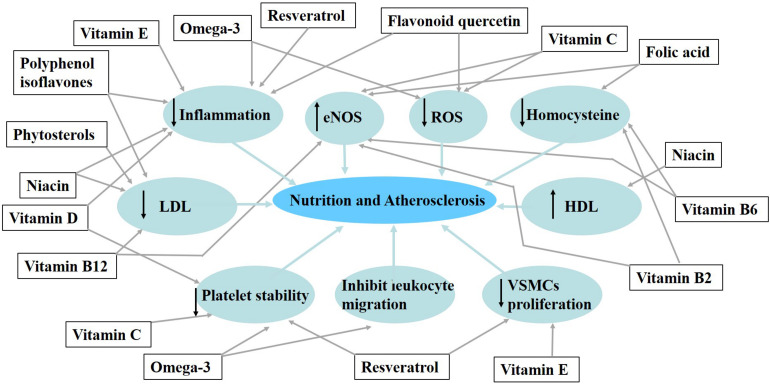
The influence of some nutrients and bioactive compounds on the process of atherosclerosis.

In this review, we summarize the nutrients that have been studied in the study of atherosclerosis. Such as phytosterols, omega-3-polyunsaturated fatty acids, polyphenol, and vitamin, we summarize the active components of nutrients in foods and their effects on atherosclerosis (Figure 1).

## Nutrient and Atherosclerosis

Diet is an inseparable part of our lives. It is generally believed that good eating habits have a certain inhibitory effect on the development of atherosclerosis. In recent years, some nutrients [such as polyunsaturated fatty acids (PUFAs), vitamins, and polyphenols], it can stabilize atherosclerotic plaque or reduce the level of biomarkers related to inflammation (Casas et al., 2018).

### Phytosterols

Phytosterols are biologically active compounds found in food, mainly derived from plants. Phytosterols can be divided into plant sterols and plant stanols. The chemical structure of phytosterols is similar to that of cholesterol. The only difference is that an extra ethyl group at the C-24 position (Gylling and Simonen, 2015), while cholesterol is only found in food which is animal origin. More than 250 types of plant sterols have been confirmed (Cohn et al., 2010). The food sources of plant sterols are mainly vegetable oils, including corn, sunflower, and soybeans, and olives, almonds, grains such as wheat germ, also fruits and vegetables, such as passion fruit, oranges, and cauliflower.

According to some guidelines and social consensus in different countries around the world (Cohn et al., 2010; Simão et al., 2013; Expert Dyslipidemia Panel of the International Atherosclerosis Society Panel Members, 2014; Gylling et al., 2014; Catapano et al., 2016). There is similar evidence that the intake of plant sterols and stanols (2 g/day) can significantly reduce the level of low-density lipoprotein-cholesterol (LDL-c) (8–10%). A study compared yogurt with phytostanol esters (4 g/day) and low-dose (2 g/day) yogurt (Vásquez-Trespalacios and Romero-Palacio, 2014). After 4 weeks of observation, it was found that it effectively reduced 10.3% LDL-c level in patients with hypercholesterolemia. A meta-analysis included 124 studies with an average phytosterol dose of 2.1 g/day (range 0.2–9.0 g/day) (Ras et al., 2014). It indicated that daily intake of 0.6–3.3 g of phytosterols, as the dose increases, LDL-c concentration gradually decreases by 6–12%.

The main mechanism for phytosterols to reduce LDL-c levels is to reduce the cholesterol absorbed through the intestine. The main mechanism is to reduce the amount of cholesterol absorbed through the intestinal lumen by competing with cholesterol to dissolve the mixed micelles in the intestinal lumen. Another mechanism is to modify the protein that encodes sterols, such as Niemann-Pick C1-like 1 (NPC1-L1) protein, to reduce cholesterol transport to intestinal epithelial cells, or intestinal epithelial cell ATP-binding cassette transporter to promote cholesterol from Intestinal epithelial cells flow out to the intestinal lumen. It can also reduce cholesterol levels through the transintestinal cholesterol excretion (Gylling and Simonen, 2015).

## Omega-3-Polyunsaturated Fatty Acids

Polyunsaturated fatty acids are straight-chain fatty acids with two or more double bonds and a carbon chain length of 18–22 carbon atoms. In the PUFA molecule, the double bond that is the most distant from the carboxyl group on the third carbon atom is called omega-3 PUFAs. The two most important unsaturated fatty acids of omega-3 PUFAs to the human body are docosahexaenoic acid (DHA) and eicosapentaenoic acid (EPA). Omega-3 PUFAs represents the most important PUFA in biology. The current research on them is mainly about their role in CVDs, inflammatory diseases, and metabolic diseases (Ander et al., 2003; Khandelwal et al., 2013; Tortosa-Caparrós et al., 2017; Schunck et al., 2018).

The REDUCE-IT trial is a randomized controlled trial that found that high-dose omega-3 PUFAs can significantly improve the prognosis of CVD, especially the use of 4 g/day of ethyl eicosapentaenoate can reduce the occurrence of cardiovascular events and reduce the risk of CVD by 25% compared with the control group (Bhatt et al., 2019). A meta-analysis conducted by Sekikawa et al. (2019) etc. included six studies, used the following criteria: adult subjects, omega-3 PUFAs (defined as ≥3.0 g/day, or 1.8 g/day in Japan), take changes in atherosclerosis as a result and perform RCT intervention time ≥6 months. Found a large dose of omega-3 PUFAs can slows down the progression of atherosclerosis and has anti-atherosclerotic effects.

Omega-3 PUFAs can regulate lipid and lipoprotein profile, and down regulate leukocyte expression and the concentration of various pro-inflammatory biomarkers related to the development of atherosclerosis. It can also reduce oxidative stress and inhibit platelets. Its activity improves the function of blood vessels (Burke et al., 2017; Innes and Calder, 2018). Studies have confirmed that in subjects suffering from MetS, supplementation with omega-3 PUFA can improve arterial vascular endothelial function and stiffness, and has parallel anti-inflammatory effects (Tousoulis et al., 2014). In an intervention study on patients waiting for carotid endarterectomy, Thies et al. (2003) found that atherosclerotic plaques can easily incorporate omega-3 PUFAs from fish oil supplements, and the induced changes can enhance atherosclerotic plaques the stability of the block. Plaque stability may be related to the reduction of non-fatal and fatal cardiovascular events caused by increased omega-3 PUFA intake. Studies have also confirmed that higher levels of EPA in plaques are associated with a decrease in the number of foam cells and T cells, leading to reduced inflammation and increased stability (Cawood et al., 2010). The results of studies also support the potential benefit of fish oil supplements in reducing the risk of atherosclerotic thrombosis in stable coronary artery disease (sCAD), and the greatest benefit for patients who have not received lipid-lowering therapy (Franzese et al., 2015).

## Polyphenol

Polyphenols are the most abundant antioxidants in the human diet and are usually found in fruits, vegetables, green tea, red wine, nuts, spices, and extra virgin olive oil. The most common polyphenols include resveratrol and flavonoids, the latter can divided into six subcategories: flavanols, flavonoids, flavanones, anthocyanins, flavonols, and isoflavones (Korakas et al., 2018). We will focus on three types of polyphenols, polyphenol isoflavones, resveratrol, and flavonoid quercetin.

### Polyphenol Isoflavones

The polyphenol isoflavones in soybeans have anti-atherosclerotic properties because their structure is similar to estrogen and binds to estrogen receptors. Consumption of soybean products can reduce the serum levels of LDL-c and triglycerides (Anderson et al., 1995). Tokede et al. (2015) analyzed a total of 35 studies (50 comparisons). The duration of treatment ranges from 4 weeks to 1 year. Ingestion of soy products resulted in a significant decrease in serum LDL-c concentration, which was −4.83 mg/dl, triglycerides was −4.92 mg/dl, and total cholesterol concentration was −5.33 mg/dl. The serum high-density lipoprotein-cholesterol (HDL-c) concentration also increased significantly, at 1.40 mg/dl, and the LDL of patients with hypercholesterolemia decreased more significantly, at −7.47 mg/dl. The results are obviously heterogeneous. However, in a community-based cross-sectional study involving 2939 subjects (2135 women and 804 men) aged 50–75 years old, it was found that greater soy consumption was related to decreased serum TC levels, dyslipidemia, hyperuricemia and less frequent cardiometabolic disorders in women (Liu et al., 2014).

Studies have shown that the isoflavones in soy protein isolate will not change the plasma cholesterol level of LDLr-null mice, but it will reduce the plasma cholesterol level of C57BL/6 mice by 30% and reduce the atherosclerotic lesion area by 50%. At the same time, studies have shown that the cholesterol clearance mechanism mediated by LDL receptors can reduce the consumption of total plasma cholesterol in C57BL/6 mice (Kirk et al., 1998). Proteomics analysis showed that soybean extract or genistein/daidzein mixture can reverse the changes in protein expression profile induced by stressors. The two application forms of isoflavones only jointly regulate protein entities related to mitochondrial dysfunction. Proteins identified through proteomics analysis indicate that soy isoflavones may enhance the anti-inflammatory response of monocytes in the blood, thereby promoting atherosclerosis prevention activities in a soy-rich diet (Fuchs et al., 2006; Wenzel et al., 2008).

### Resveratrol

Resveratrol is a natural non-flavonoid polyphenol, which is found in wine, peanuts, red wine, etc. The current research has confirmed that it has anti-oxidant (da Silva et al., 2019), anti-platelet (Bertelli et al., 1995; Baur and Sinclair, 2006), and anti-inflammatory effects (Frémont, 2000), which play an important role in the process of atherosclerosis.

A large number of epidemiological studies have reported that resveratrol can improve high blood pressure, atherosclerosis, and ischemic heart disease (Zordoky et al., 2015). A three-blind, randomized, placebo-controlled trial study found that seventy-five patients (three parallel arms) took one capsule (350 mg) per day for 6 months, which contained grape extracts rich in resveratrol, grape extract without resveratrol (similar in polyphenol content) or placebo (maltodextrin). After 6 months, only LDL-c in the GE group was reduced by 2.9% (*p* = 0.013). Conversely, LDL-c, ApoB, LDLox, and LDLox/ApoB, decreased in the Stilvid^®^ group, the ratio of non-HDL-c (total atherosclerotic cholesterol load)/ApoB increased, confirming that resveratrol reduced atherosclerosis markers and may have other cardioprotective effects beyond the gold standard drugs (Tomé-Carneiro et al., 2012). However, data indicate that resveratrol may prevent atherosclerosis in individuals who are not currently at high risk, indicating that resveratrol can be considered as the main atherosclerosis preventive agent (Agarwal et al., 2013).

The inflammatory response associated with atherosclerosis is largely regulated by the NF-κB pathway (Wang et al., 2016). NF-κB is connected with various signaling agents, which can trigger an inflammatory cascade. Animal studies have shown that the process of upregulation of SIRT-1 may have a significant impact on the activation and homeostasis of endothelial cells (Brandes, 2008; Ota et al., 2010). In endothelial cells, SIRT1 controls angiogenesis through multiple transcriptional regulators. Experimental studies have shown that the use of resveratrol can increase the serum SIRT1 concentration. Pre-treatment of human vascular smooth muscle cells (VSMCs) at a dose of 3–100 μM can significantly increase the expression of SIRT1 (Kao et al., 2010; Thompson et al., 2014). SIRT-1 inhibits NF-κB signaling pathway can inhibit the synthesis of a variety of pro-inflammatory cytokines, including: TNF-α, IL-1β, IL-6, and MCP-1.

Another important mechanism of resveratrol’s anti-atherosclerosis effect is antiplatelet activity. Its antiplatelet activity mechanism mainly focuses on inhibiting COX-1. Selective inhibition of COX-1 leads to TXA2 (thromboxane A2) synthesis reduced, which is an effective trigger for platelet aggregation (FitzGerald, 1991).

### Flavonoid Quercetin

Flavonoid quercetin is an important food antioxidant, found in vegetables and fruits, especially onions, apples and berries, wine and tea. The quercetin can be used as a valuable protective agent in a variety of diseases including cardiovascular inflammatory diseases (D’Andrea, 2015). Quercetin can prevent obesity induced by high-fat diet, and its anti-obesity effect may be related to the regulation of adipogenesis at the transcriptional level (D’Andrea, 2015). A randomized, double-blind, placebo-controlled crossover trial involving 37 apparently healthy (hypertensive) hypertensive men and women (40–80 years old) confirmed that quercetin may provide the heart by improving endothelial function and reducing inflammation protective effects (Dower et al., 2015). A meta-analysis of randomized controlled trials (RCT) showed that supplementation with quercetin had a significant effect on C-reactive protein-especially in patients with doses greater than 500 mg/day and CRP <3 mg/l. Other polyphenols, such as cocoa flavanols, have been found to improve endothelial function and protect the cardiovascular system (Lin et al., 2016; Mozaffarian and Wu, 2018).

Studies have found that the polyphenol flavonoid quercetin reduces the inflammatory response induced by high cholesterol levels and regulates the inflammatory process of atherosclerosis by affecting the TLR-NF-κB signaling pathway. In addition, the increase of quercetin can significantly reduce serum inflammatory mediators expression of mRNA such as COX, 5-LOX, MPO, CRP, and NOS (Bhaskar et al., 2016). Thereby reducing the atherosclerosis process related to endothelial dysfunction. Mechanism studies have shown that dietary hyperquercetin can significantly reduce the expression of p47phox in the aorta of ApoE−/− mice fed a high-fat diet and inhibit NADPH oxidase-derived oxidative stress, however, the expression and activity of the antioxidant enzyme heme oxygenase-1 (HO-1) are enhanced. *In vitro*, quercetin significantly reduced the formation of NADPH oxidase-derived O2⋅-in endothelial cells by inducing HO-1. It is proved that quercetin has indirect antioxidant properties in the process of atherosclerosis with NADPH oxidase and HO-1 (Luo et al., 2020).

## Vitamin

The diet involved in some micronutrients in atherosclerosis role is recognized. Vitamins, in particular. Studies demonstrate that increase the intake of vitamins in patients with subclinical atherosclerosis can reduce and slow down the incidence of CVD events, thereby preventing the development of pathological events (Hansson et al., 2006). However, with the population ages and the diet diversifies, vitamin deficiency is not uncommon in the worldwide, which may be an additional explanation for the increasing rate of coronary heart disease and cerebrovascular disease caused by atherosclerosis. In order to further raise people’s awareness, it is necessary to further emphasize the influence of vitamins on the development of atherosclerosis in order to better guide the prevention of CVD.

The current new research focuses on the effect of vitamins on atherosclerosis. Especially the antioxidant capacity, so that the damage can still be reversed or at least slowed down, thereby preventing or slowing down the occurrence of vascular events caused by atherosclerosis. It appears that vitamins which with antioxidant and anti-inflammatory properties, may play an important role in targeting the subclinical atherosclerosis by equilibrating the balance of oxidation and antioxidation in human metabolism (Apryatin et al., 2018). Different groups of vitamins may play different roles, such as improving endothelial function, improving metabolism, inhibiting the renin-angiotensin-aldosterone system, anti-inflammatory, antioxidant, lowering blood homocysteine acid levels, and reversal of arterial calcification. Serum Vitamin B, C, D, E levels are of great significance for assessing cardiovascular risk and early prevention of CVD.

## Vitamin E

Vitamin E is a type of vitamin with antioxidant effect and an essential micronutrient. It exists in plants, seeds and their derivatives. It contains eight different isomers: four tocopherols (T) (α, β, γ, and δ) and four tocotrienols (T3) (α, β, γ, and δ). Among them, tocopherol α-T is the most active isomer of vitamin E. It is precursor-free and has important antioxidant effects (Parker et al., 1993). A lot of research has been done on the prevention of atherosclerosis. Vitamin E appears to be found in fat deposits, lipoproteins, and lipid-rich tissues. It seems to be involved in multiple stages of inflammation and immunity by regulating cell functions and gene expression (Rasool et al., 2008). By eliminating oxygen freelance fundamentals in cells, tissues, or membranes, it protects CVD, metabolic disorders, and other diseases at risk of oxidative stress (Meganathan and Fu, 2016).

Several interventional studies have shown that the effect of vitamin E in the early subclinical atherosclerosis phase of atherosclerosis seems encouraging. A study that used vitamin E and placebo to intervene in 36 healthy men to assess arterial compliance by measuring pulse wave velocity (PWV) and enhancement index (AI) concluded that vitamin E supplementation for 2 months had a tendency to improve arterial compliance (Waniek et al., 2017). A population-based study of the effects of antioxidant vitamin supplementation in preventing atherosclerosis has shown for the first time that a reasonable dose of a combined dose of vitamin E and vitamin C can delay the progression of common cervical atherosclerosis in men (Rasool et al., 2008). This may be further evidence of the role of vitamins in atherosclerosis trials revealed that patients with subclinical atherosclerosis treated with vitamin E a significant improvement in peripheral-artery disease and a reduced incidence of pectoris. This may imply that the earlier prevention, even if the progression of atherosclerosis is not fully controlled, can delay the occurrence of CVD. Many observational and cohort studies suggested a negative association between dietary vitamin E supplementation intake and CV events (Mozos et al., 2017). However, a meta-analysis has shown that high doses of vitamin E have oxidative effects, when vitamin E dose ≥400 IU may increase the all-cause mortality rate. Therefore, the benefits of vitamin E supplementation need to be accompanied by consideration of the hazards of higher doses.

Vitamin E inhibits the expression of adhesion molecules on endothelial cells and ligands on monocytes, and reduces the adhesion interaction between them. This is an important early event that can trigger the formation of fat streaks and atherosclerosis (Devaraj et al., 1996; Freedman et al., 1996; Wu et al., 1999). It can regulate inflammation by inhibiting 5-lipoxygenase, thereby reducing the release of interleukin-1β released by monocytes. It may also reduce the adhesion of monocytes *in vitro* by inhibiting the activation of nuclear factor NF-κB (Devaraj and Jialal, 1998). α-tocopherol inhibits protein kinase C (PKC)-mediated monocyte superoxide production, SMC proliferation, and platelet aggregation and adhesion (Ricciarelli et al., 1998; Keaney et al., 1999). Studies also show that vitamin E, through its non-antioxidant properties, may inhibit smooth muscle cell proliferation (Ricciarelli et al., 1998) and platelet aggregation (Freedman et al., 1996), which are important processes in plaque formation and atherosclerosis.

## Vitamin C

Vitamin C is a polyhydroxy compound. The two adjacent enol hydroxyl groups at the 2nd and 3rd positions in the molecule are easily dissociated to release H+, so it has acid properties and is also called ascorbic acid. Dietary sources of vitamin C are widely found in fresh vegetables and fruits, such as tomatoes, cauliflower, bell peppers, dark leafy vegetables, bitter gourd, citrus, grapefruit, grapes, kiwi, oranges, etc. Vitamin C must be obtained from external sources (primarily fruits and vegetables) because humans cannot synthesize it internally.

A meta-analysis consisting of 13 independent cohorts included 278,459 people (including 9143 CHD events). They were followed up for an average of 11 years and found that the daily intake of fruits and vegetables increased from less than three to more than five. A 17% reduction in heart disease risk is related (He et al., 2007).

Previous studies have demonstrated that vitamin C being able to scavenge ROS. It may prevent the oxidation of LDL-c by reducing the α-tocopherol free radical. Inhibiting ROS-mediated direct damage to the vascular endothelium and oxidative stress-induced signaling pathways, which are involved in the occurrence and development of atherosclerosis (Haendeler et al., 1996). Therefore, vitamin C plays an important role in preventing atherosclerosis and delaying the progression of coronary heart disease. Vitamin C can also prevent activation of platelets and apoptosis. A study investigated the relationship between vitamin C in supplements and early atherosclerosis [detection of carotid intima-media thickness (IMT)] and found that vitamin C in food is associated with accelerated early atherosclerosis measured by carotid IMT (Meganathan and Fu, 2016). Many studies have shown that atherosclerosis is negatively related to the intake of antioxidants, in contrast, some clinical trials have found that vitamin C are not beneficial for supplementary treatment of CVD. However, these clinical trials are not without their limitations. Antioxidant therapy for a relatively short period of time or treatment of patients with advanced disease may not provide information related to disease.

## Vitamin D

Vitamin D is a group of steroids, the most important of which are vitamin D3 (cholecalciferol) and vitamin D2 (ergocalciferol). They can be obtained from diet and various supplements. The human body can also synthesize vitamin D.

Vitamin D affects many cells involved in atherogenesis, such as immune cells, endothelial cells, smooth muscle cells, and cardiomyocytes (Brewer et al., 2011). Vitamin D may influence the pathophysiology of atherosclerosis through decreasing the expression of TNFα, IL-6, IL-1, and IL-8 in isolated blood monocytes (Giulietti et al., 2007; Kassi et al., 2013). It may regulate the expression of thromboregulatory proteins and tissue factors in monocytes, thereby affecting platelet aggregation and thrombosis activity (Koyama et al., 1998). Thereby possibly preventing luminal rupture and thrombosis due to plaque instability (Oh et al., 2009). In addition, there is conclusive evidence that VDR and 1α-hydroxylase are expressed in the heart and blood vessels. Vitamin D has been shown to delay the development of porcine coronary artery disease by inhibiting NF-κB activation, supporting that vitamin D is considered an important factor in CVD (Chen et al., 2016).

## Vitamin B

Vitamin B is an essential nutrient for all human tissues. It is all coenzymes and participates in the metabolism of sugar, protein and fat in the body, so it is classified as a family. There are more than twelve types in this category, nine of which are considered essential vitamins for the human body, all of which are water-soluble vitamins. They stay in the body for only a few hours and must be supplemented daily. Folate (B9), B12, B6, niacin (B3), and riboflavin (B2) in B vitamins are believed to be related to reducing the risk of CVD.

### Niacin

Niacin, also known as vitamin B3, or vitamin PP, is a water-soluble vitamin belonging to the vitamin B family. Niacin is converted into nicotinamide in the human body. Niacinamide is a component of Coenzyme I and Coenzyme II and participates in lipid metabolism in the body. Food-derived niacin is widely found in arterial liver, lean meat, cereals, beans, and green leafy vegetables. In addition to being directly ingested from food, niacin can also be converted from tryptophan in the body, with an average of about 60 mg tryptophan converting 1 mg niacin.

In pharmacological doses, niacin can reduce serum levels of LDL-c, very VLDL-c and lipoprotein (a) (Lp a). In addition, it can significantly increase the serum level of HDL-c. The mechanism of niacin to lower blood lipids and prevent atherosclerosis may have the following two aspects. Niacin can up-regulate PPARγ by stimulating the ATP-binding cassette transporter A1 in monocytes and macrophages, and ultimately lead to reverse cholesterol transport (Rubic et al., 2004). On the other hand, niacin can inhibit inflammation to prevent atherosclerosis. An animal study showed that niacin inhibits vascular inflammation by down-regulating the NF-κB signaling pathway (Si et al., 2014). Niacin lowers can reduce the level of CRP and lipoprotein-related phospholipase A2 (an independent risk factor for CVD) (Kuvin et al., 2006). Studies have shown that niacin has been shown to inhibit the expression and release of chemokine induced by TNF-α (Digby et al., 2010).

### Folic Acid

Folic acid is a water-soluble vitamin. The biologically active form of folic acid is tetrahydrofolate. Human intestinal bacteria can synthesize folic acid, which can cause folic acid deficiency when malabsorption, metabolic disorders or long-term use of intestinal antibacterial drugs. In addition, folic acid is also widely present in animal and plant foods, rich in: offal, eggs, fish and pears, broad beans, beets, spinach, cauliflower, celery, citrus, nuts, and soy foods.

### Vitamin B12

Vitamin B12, also called cobalamin, is the only vitamin that needs the help of intestinal secretions (endogenous factors) to be absorbed. Vitamin B12 in nature is mainly synthesized by bacteria in the rumen and colon of herbivores. Therefore, its dietary sources are mainly animal foods. Among them, animal offal, meat, and eggs are rich sources of vitamin B12. Soy products will produce part of vitamin B12 after fermentation. Human intestinal bacteria can also synthesize part of it.

### Vitamin B6

Vitamin B6 is also known as pitocin, which includes pyridoxine, pyridoxamine, and pyridoxal. It exists in the form of phosphate ester in the body and is a water-soluble vitamin. Vitamin B6 is higher in meat, cereal products (especially wheat), vegetables and nuts. Vitamin B6 is a component of some coenzymes in the human body and participates in various metabolic reactions, especially closely related to amino acid metabolism.

### Vitamin B2

Vitamin B2, also called riboflavin, is slightly soluble in water and stable when heated in neutral or acidic solutions. It is a component of the prosthetic group of yellow enzymes in the body (yellow enzymes play a role of hydrogen transfer in biological redox). The storage of vitamin B2 in the body is very limited, so it must be provided by the diet every day. Vitamin B2 is widely present in various foods, but the content of animal foods is usually higher than that of plant foods, such as liver, kidney, heart, egg yolk, eel, and milk of various animals. Many green leafy vegetables and legumes are also high in content, while cereals and general vegetables are low in content.

Current studies have found that B vitamins can reduce homocysteine levels. Homocysteine can be methylated into methionine. Vitamin B12 and riboflavin are used as cofactors in this process. At the same time, folic acid can provide methyl groups to react. It can also be metabolized into cysteine with the help of vitamin B6 and removed from the circulation. In recent years, more and more studies have shown that high plasma concentration of homocysteine, enhances the risk of atherothrombotic vascular disease (Knapen et al., 2015; Fulton et al., 2016). High homocysteine levels are an independent risk factor for atherosclerosis. It can increase oxidative stress and vascular smooth muscle growth and can also cause endothelial cell damage (Mudd, 1985). Both folic acid and B12 are necessary components for the conversion of HCY to methionine and are significantly negatively correlated with HCY levels. It was found in hemodialysis patients that combined folic acid and vitamin b12 supplementation can reduce the degree of atherosclerosis (McCully, 1969). Although studies have confirmed that vitamin B12 and folic acid can reduce plasma high homocysteine levels and play a role in protecting atherosclerosis (Shargorodsky et al., 2009), the specific efficacy of preventing CVD remains to be further studied. But for patients with an increased risk of atherosclerosis, it is necessary to monitor serum B vitamins and appropriate supplements.

In conclusion, we have summarized the relevant research and possible mechanisms of nutrients in the occurrence and progression of atherosclerosis (Table 1). In this review, we outline the current research on nutrients in atherosclerosis and their effects on related diseases.

**TABLE 1:** Proposed mechanism of nutrients in preventing atherosclerosis.

		**↓Inflammation**	**↓ROS**	**↓Homocysteine**	**↓LDL**	**↑HDL**	**↓Platelet stability**	**Inhibit leukocyte migration**	**↓VSMCs proliferation**	**↑eNOS**
Phytosterols					•					
Omega-3		•	•				•	•		
Polyphenol isoflavones		•			•					
Resveratrol		•					•		•	
Flavonoid quercetin		•	•							
Vitamin E		•							•	
Vitamin C			•				•			•
Vitamin D		•					•			
Vitamin B	Niacin	•			•	•				
	Folic acid			•						•
	Vitamin B12			•						•
	Vitamin B6			•						•
	Vitamin B2			•						•

## Conclusion

Atherosclerosis is one of the most important risk factors for CVD and stroke. At present, clinical doctor reducing the level of LDL-c delays the development of atherosclerosis. In recent years, some studies have emphasized the important role of good eating habits and living habits in the development of atherosclerosis. Including phytosterols, omega-3-polyunsaturated fatty acids, polyphenol, and vitamin, which mentioned in this article, can directly or indirectly act on the vascular system by reducing inflammation, reducing oxidative stress, or forming active metabolites. We need to pay attention to the nutritional status of patients with atherosclerosis, which is a controllable risk factor for atherosclerosis, which can improve the patient’s nutritional status and thereby improve the prognosis of patients. Therefore, it is important to establish the consensus on nutrient intake in the field of nutrition to reduce the occurrence of atherosclerosis, thereby reducing the incidence of cardiovascular and cerebrovascular diseases. Diet is an inseparable part of our lives, and whether the nutrients in food can benefit our body is worthy of long-term research in the future.

## Author Contributions

GC summarized the figure. All authors listed have made a substantial, direct and intellectual contribution to the work, and approved it for publication.

## Conflict of Interest

The authors declare that the research was conducted in the absence of any commercial or financial relationships that could be construed as a potential conflict of interest.
